# Human brain organoids: an innovative model for neurological disorder research and therapy

**DOI:** 10.3389/fncel.2025.1658074

**Published:** 2025-08-26

**Authors:** Hancheng Li, Junxiao Zhu, Jieyu Li, Yangkai Wu, Chaohua Luo, Yuting Huang, Jieru Wu, Wenhua Liu, Hongwu Wang, Zhixian Mo

**Affiliations:** 1 Department of Pharmaceutical Engineering, School of Food and Pharmaceutical Engineering, Zhaoqing University, Zhaoqing, China; 2 Department of Pharmacology of Chinese Medicine, School of Traditional Chinese Medicine, Southern Medical University, Guangzhou, China; 3 Key Laboratory for Research and Utilization of Southern Medicine, Zhaoqing University, Zhaoqing, China; 4 Risk Assessment Laboratory for Agricultural Product Quality and Safety, Ministry of Agriculture and Rural Development, Zhaoqing University, Zhaoqing, China

**Keywords:** human brain organoids, neurological disorders, disease modeling, therapeutic innovation, induced pluripotent stem cells

## Abstract

The emergence of human brain organoids (hBOs) has transformed how we study brain development, disease mechanisms, and therapy discovery. These 3D *in vitro* neural models closely mimic the cellular diversity, spatial structure, and functional connectivity of the human brain, providing a groundbreaking platform that outperforms traditional 2D cultures and animal models in studying neurodevelopment and neurological disorders. To further explore the potential of hBOs technology, we review current literature focusing particularly on its applications for diagnosing and treating major neurological diseases such as Alzheimer’s disease, Parkinson’s disease, and other related neurological disorders. Using patient-derived induced pluripotent stem cells combined with cutting-edge gene-editing technologies, hBOs enable highly precise mechanistic studies and scalable drug screening. Moreover, we further discuss the advantages and current limitations of hBOs. Despite these challenges, hBOs remain a transformative platform for the development of targeted neurotherapeutics. Collectively, this review offers a solid foundation for advancing neuroscience research and fostering innovative treatment strategies for neurological disorders.

## 1 Introduction

The human brain is characterized by exceptional cellular diversity and intricate synaptic architecture, presenting considerable challenges for modeling neurological disorders (ND) such as Alzheimer’s disease (AD) and autism spectrum disorders (ASD). Although traditional two-dimensional (2D) cell cultures and animal models have significantly advanced neuroscience research (Wang Z. et al., 2017), they fail to replicate the human brain’s three-dimensional (3D) structure and species-specific features, limiting their translational relevance. Organoid technology, first developed in cancer research in 1946, gained new momentum with the advent of pluripotent stem cell (PSC) technologies in 1998. These breakthroughs enabled the generation of organoids resembling organ-specific structure and function across various systems, including the brain, liver, gut, and kidney (Mishra et al., 2024). In particular, human brain organoids (hBOs), derived from human pluripotent stem cells (hPSCs) such as embryonic stem cells (ESCs) and induced pluripotent stem cells (iPSCs), have the capacity to self-organize into 3D structures that recapitulate key features of the human brain (Lee et al., 2017).

**FIGURE 1:**
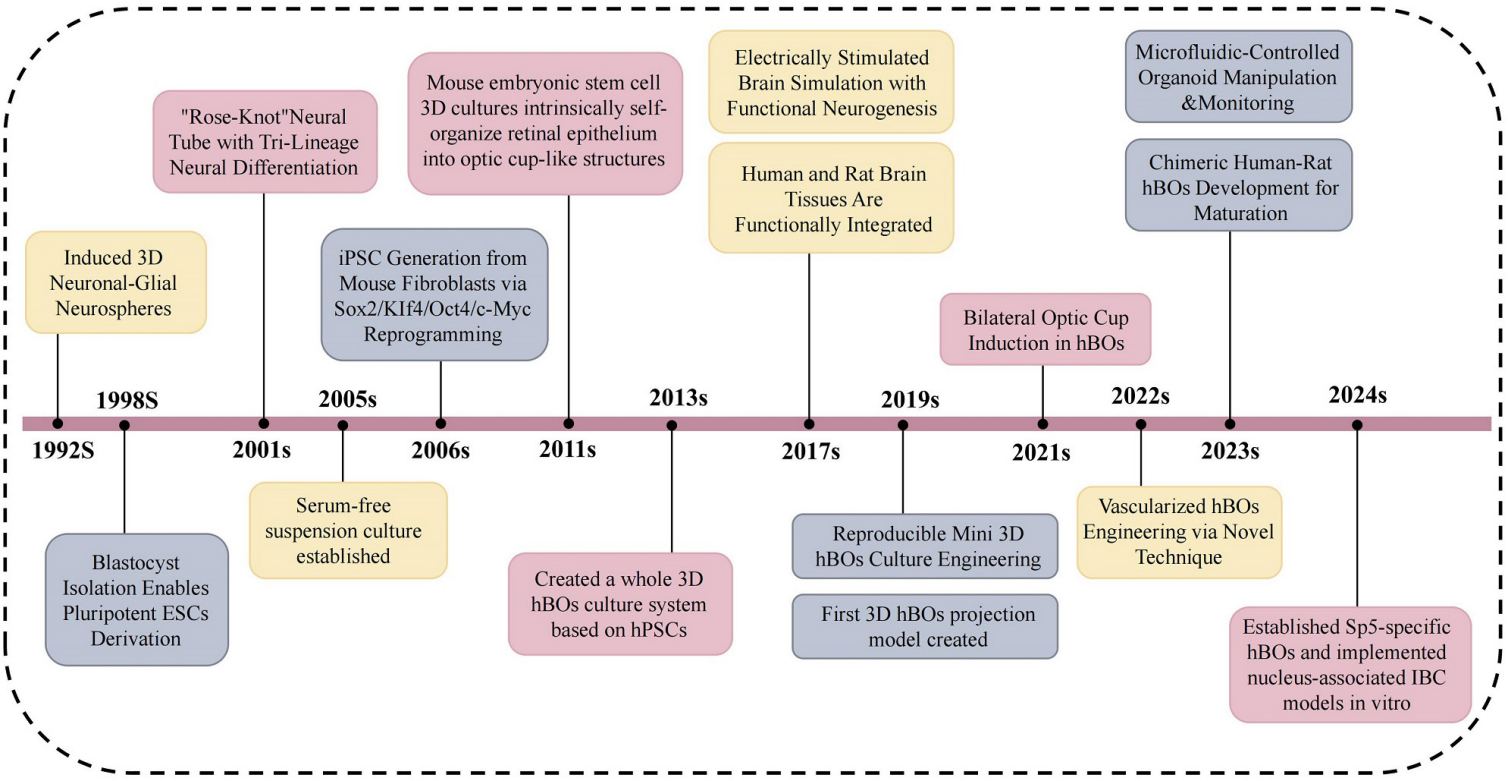
Illustrates major milestones in hBOs research from 1992 to 2024. Yellow denotes progress in culture optimization and functional maturation, enabling the shift from simple neurospheres to functional organoids. Gray indicates innovations in methodology, including reprogramming, engineered platforms, and novel disease models. Pink marks advances in morphogenesis and cell lineage specification, from neural tube-like structures to region- or subtype-specific hBOs. The timeline emphasizes trilineage differentiation, emergence of complex models such as Sp5 and IBC, and enhanced neural circuit formation. This three-decade evolution highlights hBOs as powerful *in vitro* systems for neuroscience. Sp5, spinal trigeminal nucleus; IBC, inter-brain connection.

Since 1992, continuous improvements in hBOs have significantly expanded their applications in neuroscience (Figure 1). These models have become essential tools for studying human-specific mechanisms of brain development and neuropathology (Kwak et al., 2024). Compared with 2D cultures and animal models, hBOs demonstrate superior fidelity in replicating human brain architecture, offering broad utility in disease modeling, drug screening, and personalized medicine. However, several limitations persist, including restricted vascularization, inter-organoid heterogeneity, and unresolved ethical concerns (Kanupriya et al., 2025). These factors often lead to hypoxic core regions, increased cellular stress, and limited capacity to model late-stage ND. Moreover, the absence of inter-organ interactions restricts their utility in capturing the systemic complexity of disease pathogenesis. Addressing these challenges is crucial for enhancing the biological fidelity and clinical relevance of hBO-based platforms.

## 2 hBOs modeling the neural microenvironment

**FIGURE 2:**
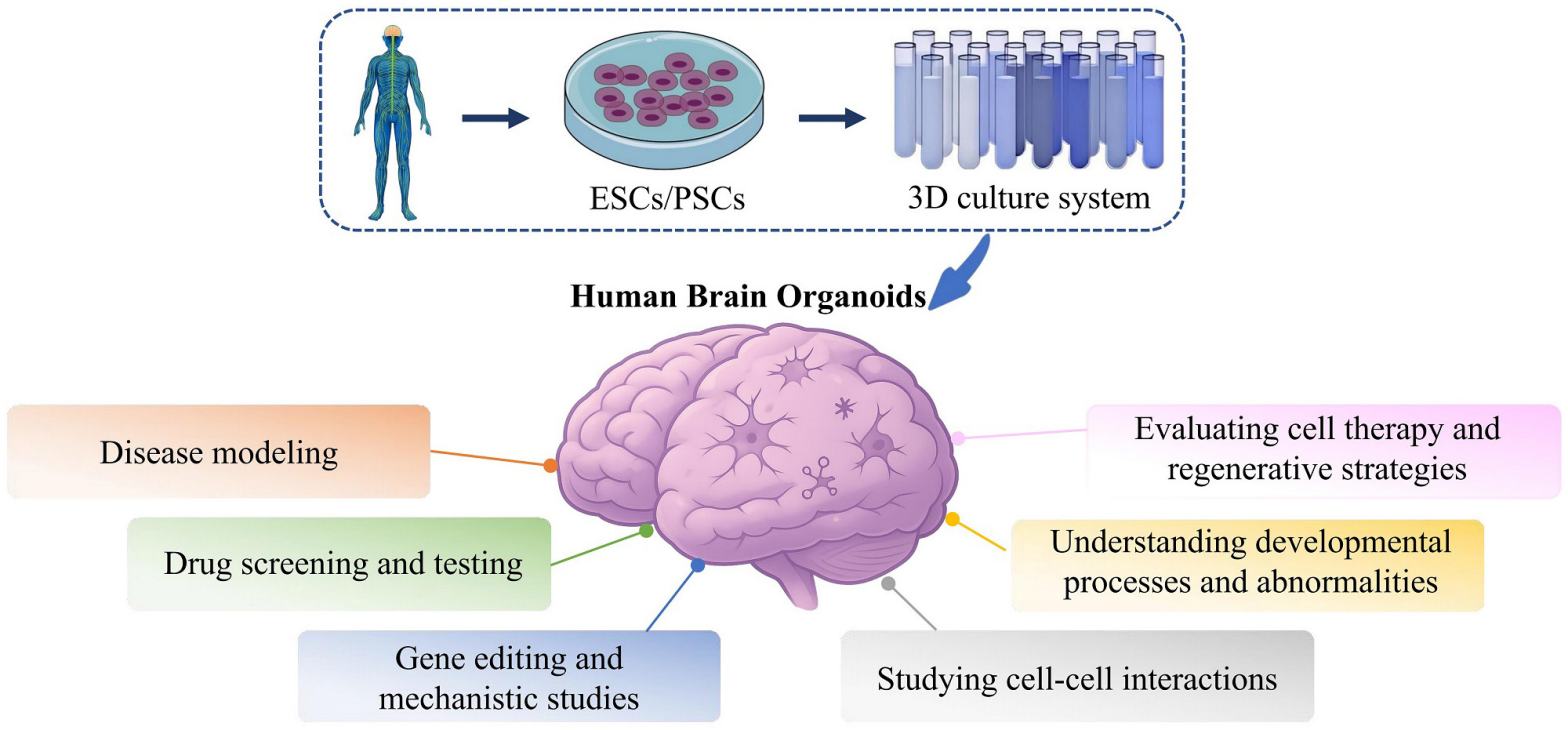
Applications of hBOs in investigating disease mechanisms and conducting drug screening. hBOs are a novel *in vitro* modeling platform for neurological disorder research and drug development. Using these models, researchers establish disease-specific hBOs, conduct high-throughput drug screening, and employ molecular biology and imaging for phenotype analysis. hBOs effectively replicate disease pathology, demonstrate strong predictability and reproducibility in drug testing, and reveal new molecular insights into disease mechanisms. Embryonic stem cells, ESCs; Pluripotent stem cells, PSCs.

Beyond replicating brain structure, human brain organoids (hBOs) closely model the dynamic neural microenvironment. Unlike traditional 2D cultures and animal models, hBOs cultured in 3D systems can produce extracellular matrices (ECMs) that support autocrine and paracrine signaling, thereby enabling more physiologically relevant modeling of cellular proliferation, migration, and differentiation (Mimeault et al., 2007; Acharya et al., 2024). It is important to note that the development and maintenance of hBOs often rely on artificial ECMs such as Matrigel and Geltrex. These materials are commonly used to embed organoids or are included in culture media to provide essential structural support and promote proper tissue organization. Through guided differentiation of iPSCs, hBOs develop structural and functional features resembling early human neural tissue (Qian et al., 2019). Recent advances such as incorporation of vascular-like networks and extended culture stability have improved physiological accuracy (Sun et al., 2022). These enhancements support applications in neurodevelopmental research, disease mechanism elucidation, and therapeutic screening. To better capture complex disease phenotypes, recent bioengineering strategies aim to integrate neuroimmune components, enable *in vivo* transplantation, and construct multi-regional or multi-organ systems. These innovations improve systemic modeling capabilities and enhance translational relevance. With continued progress in bioengineering and multi-omics integration, hBOs are becoming indispensable tools for neuroscience research, disease modeling, and regenerative medicine (Figure 2).

## 3 Unguided versus guided hBOs in ND modeling

Human brain organoids have become indispensable tools for modeling ND, with a key methodological distinction being the use of unguided or guided differentiation strategies. Unguided hBOs rely on spontaneous self-organization of pluripotent stem cells (PSCs) without exogenous patterning signals, resulting in the generation of heterogeneous brain regions such as the forebrain, midbrain, and hindbrain within a single organoid (Lancaster et al., 2013). This approach recapitulates early brain development and is suitable for modeling disorders such as microcephaly, Zika virus infection, and cortical malformations (Qian et al., 2019). However, unguided organoids suffer from batch variability, inconsistent regional identity, and stochastic architecture, limiting their reproducibility and suitability for region-specific disease modeling and high-throughput drug screening (Velasco et al., 2019).

In contrast, guided hBOs are derived by applying defined patterning cues to direct differentiation toward specific brain regions, such as the cortex, midbrain, or hypothalamus (Qian et al., 2016; Jo et al., 2016). This strategy enhances regional fidelity, reproducibility, and experimental control. For example, midbrain hBOs enriched with dopaminergic neurons are used to model Parkinson’s disease (PD) (Smits and Schwamborn, 2020), while cortical organoids facilitate the study of amyloid and Tau pathologies in AD (Raja et al., 2016). Nevertheless, guided organoids may oversimplify the native brain environment and often lack inter-regional connectivity, limiting their utility in modeling network-level dysfunctions. To address these limitations, hybrid models such as “assembloids” have been developed, fusing region-specific organoids to recreate inter-regional interactions (Bagley et al., 2017). Future advances in single-cell multi-omics, spatial transcriptomics, and bioengineering are expected to integrate the strengths of both approaches, improving the physiological relevance, reproducibility, and translational value of hBO-based models in ND research.

## 4 hBOs × multi-omics represent a novel strategy

Human brain organoids replicate key structural and functional features of the brain and, when combined with multi-omics technologies including transcriptomics, proteomics, and epigenomics, offer a powerful strategy to decode mechanisms of ND (Taglieri et al., 2025). Single-cell RNA sequencing (scRNA-seq) reveals cell-type heterogeneity and disease-relevant gene expression patterns, such as Wnt signaling disruptions in ASD (Kiaee et al., 2021) and neuroinflammatory markers in AD (Abdelbasset et al., 2024). Because transcript levels do not always reflect protein abundance or activity, proteomics provides essential complementary insights. Mass spectrometry-based profiling can identify post-translational modifications such as phosphorylation, as demonstrated by tau hyperphosphorylation in AD organoids, which is a hallmark of altered signaling pathways (Bracha et al., 2024; Marinho et al., 2023). Epigenomic approaches, including ATAC-seq and DNA methylation analysis, provide information on chromatin accessibility and transcriptional regulation. In AD hBOs, ATAC-seq has revealed reduced enhancer activity in genes associated with neuronal apoptosis, corroborating transcriptomic findings (Marinho et al., 2023). Integrating scRNA-seq with proteomic and phosphoproteomic data improves understanding of transcriptional and post-transcriptional regulation (Pieters et al., 2021), and enables tracking of critical pathways such as NF-κB and PI3K-Akt in disease models (Tanaka, 2024). These multi-omics strategies expand hBOs from static structural models into dynamic systems for mechanistic insight, biomarker discovery, and drug development.

## 5 Applications and innovations of hBOs in ND research and therapy

Human brain organoids offer enhanced physiological relevance by mimicking the 3D architecture and cellular microenvironment of the human brain. When derived from patient-specific iPSCs, hBOs can reproduce disease-specific phenotypes and provide insights into cell-cell interactions, circuit-level dynamics, and developmental processes underlying ND (Hartlaub et al., 2019). Compared to conventional 2D cultures, which lack spatial complexity and cellular diversity (Lancaster and Knoblich, 2014), hBOs enable more accurate modeling of complex neural processes and offer a robust platform for translational research. As a result, hBOs are increasingly employed in ND research due to their adaptability and biological fidelity (Castiglione et al., 2022; Zhou et al., 2023). Recent advances, including CRISPR/Cas9-based gene editing (Driehuis and Clevers, 2017) and astrocyte-enriched protocols (Bagley et al., 2017), have expanded the scope of mechanistic studies, particularly in modeling neuron-glia interactions and evaluating targeted therapies.

To contextualize the advantages of hBOs, we compared five commonly used neural modeling systems (Table 1), highlighting the superior structural fidelity and translational potential of hBOs. Their ability to reproduce the 3D cytoarchitecture of native brain tissue and capture human-specific features makes them invaluable for high-throughput screening and disease modeling. Nevertheless, batch-to-batch variability caused by differences in stem cell source, reagent quality, and manual handling remains a critical challenge (Humpel, 2015; Quadrato et al., 2017). Other models such as animal chimeras (Bourret et al., 2016), brain-on-a-chip platforms (Amirifar et al., 2022; Kogler et al., 2023), and 2D cultures (Takahashi and Yamanaka, 2006; Zhang et al., 2013) offer unique advantages in terms of scalability or in vivo relevance, but fail to replicate the full complexity of human neurobiology. Standardization in quality control, biomarker-based assessment, and scalable production pipelines is essential to overcome these limitations and improve reproducibility.

**TABLE 1:** Summary of key advantages and limitations of three commonly used neural modeling platforms: 3D human brain organoids (hBOs), animal chimera, brain-on-a-chip systems, 2D cultures/primary neurons, and 2D iPSC-derived neurons.

Models	Advantages	Limitations
3D hBOs (Humpel, 2015; Quadrato et al., 2017)	– Mimic the complex structure and microenvironment of native brain tissue - Enable realistic cell–cell interactions and molecular transport – Accurately represent patient-specific disease phenotypes using iPSC-derived cells – Contain multiple neural cell types including neurons and glia	– Insufficient nutrient diffusion for long-term culture – Absence of all relevant cell types and microvascular systems – Technically complex and unstable; lack of standardized, scalable protocols – Donor genetic variability may introduce phenotypic inconsistency
Animal chimeras (Bourret et al., 2016)	– *In vivo* integration and long-term studies – Includes vascular and immune context – Suitable for behavioral assessment	– Ethical and legal concerns – Variable engraftment – Requires immunosuppression
Brain-on-a-chip systems (Amirifar et al., 2022; Kogler et al., 2023)	– Precisely controlled environment – Real-time signal monitoring – Reproducible and scalable	– Limited tissue complexity – Lacks 3D architecture – High technical cost
2D cultures/primary neurons (Takahashi and Yamanaka, 2006)	– Simple, well-controlled experimental systems – Suitable for high-throughput screening and large-scale studies – Can exhibit clear phenotypic responses to drugs	– Lack of 3D architecture and physiological relevance – Limited modulation of culture microenvironment – Poor representation of human brain complexity
2D iPSC-derived neurons (Zhang et al., 2013; Quadrato et al., 2017)	– Generate functional neurons from patient-specific iPSCs – Detectable phenotypes that inform drug efficacy and disease mechanisms	– Lack 3D spatial organization – Absence of multicellular interactions impairs neuronal function and network modeling

This comparison highlights the physiological relevance, technical feasibility, and experimental utility of each model system.

**FIGURE 3:**
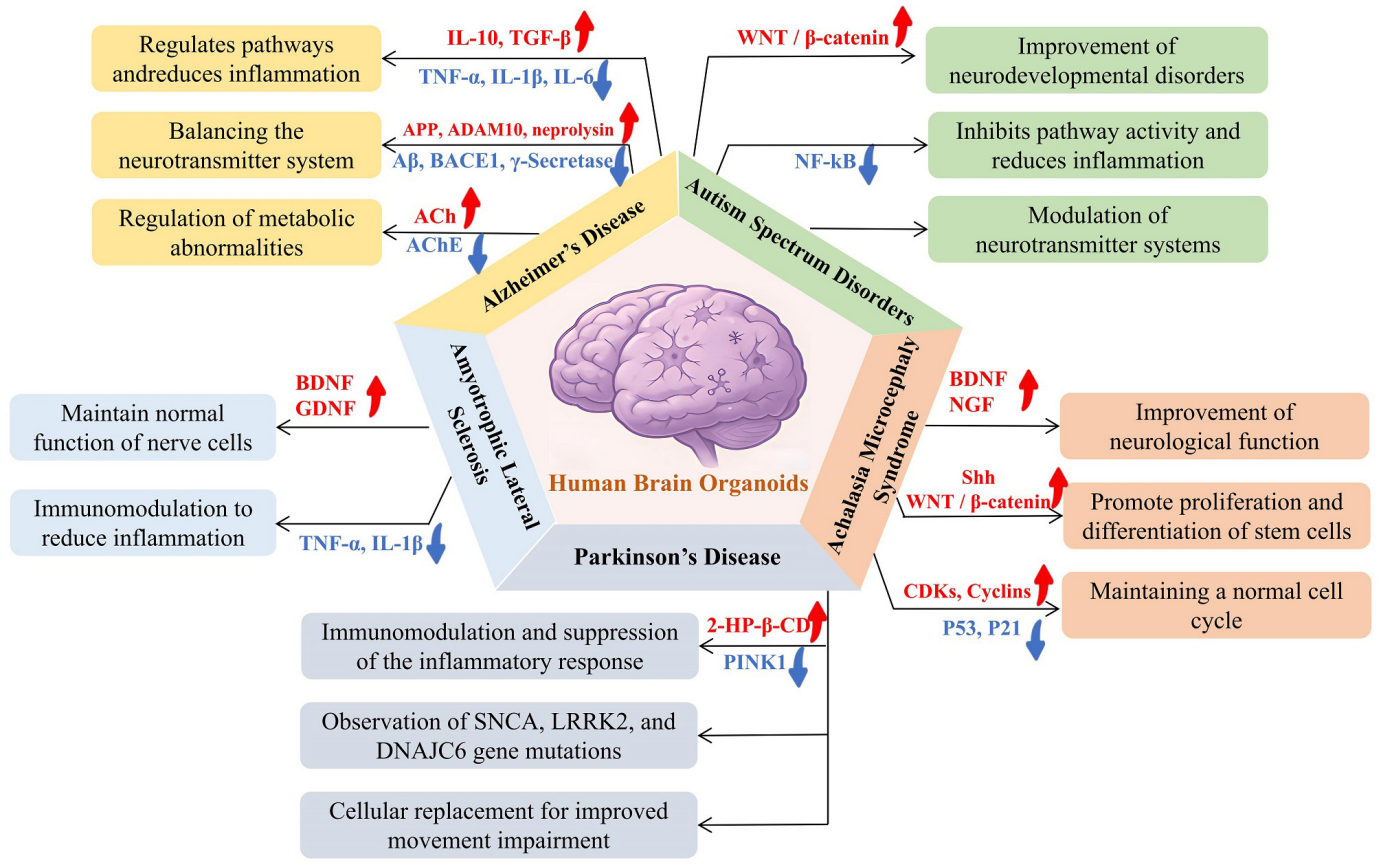
Applications of hBOs in modeling disease mechanisms across five common neurological disorders: Alzheimer’s disease (AD), autism spectrum disorder (ASD), achalasia microcephaly syndrome (AMS), Parkinson’s disease (PD), and amyotrophic lateral sclerosis (ALS). The research demonstrated specific applications in disease modeling, including simulating beta-amyloid plaque and tau tangles in AD, studying neurodevelopmental abnormalities and synaptic dysfunction in ASD, observing brain developmental disorders and microcephaly in AMS, recapitulating dopaminergic neuron degeneration in PD, and modeling motor neuron degeneration and neuroinflammation in ALS. The hBOs provide a valuable *in vitro* model system for investigating the pathogenesis of neurological diseases, capable of recapitulating key pathological features and aiding in disease mechanism research and drug development.

Despite their promise, hBOs still face major limitations, including the lack of functional vasculature, mature microglia, and complete neural circuitry (Bao et al., 2021; Cao et al., 2023). Furthermore, conventional monoculture hBOs do not replicate systemic inter-organ communication, an increasingly recognized contributor to ND pathogenesis. To overcome these issues, several innovative strategies have been developed (Table 2). Incorporation of microglia enables the establishment of neuroimmune models simulating brain-specific immune responses (Ao et al., 2021; Sabate-Soler et al., 2022; Samudyata et al., 2022). Transplantation of hBOs into rodent brains supports *in vivo* maturation and therapeutic validation (Dong et al., 2021; Revah et al., 2022), while multi-region organoid fusion approaches (assembloids) allow for the study of interregional connectivity and systemic interactions (Kasai et al., 2020; Zhu et al., 2023). These advances have significantly improved the biological and translational relevance of hBO-based platforms. hBOs can now model a broad range of disorders, including AD, PD, amyotrophic lateral sclerosis (ALS), autism spectrum disorder (ASD) and achalasia-microcephaly syndrome (AMS), faithfully reproducing hallmark pathologies *in vitro* (Figure 3). As evidence mounts that many ND originate from early developmental disruptions (Barnat et al., 2020; Yeh et al., 2018), and with a rising incidence of early-onset neurological symptoms in younger populations (Jia et al., 2023), the utility of hBOs in modeling disease mechanisms and personalizing pharmacological interventions becomes increasingly evident. With continued refinement, hBOs are poised to become cornerstone tools in precision neurology.

**TABLE 2:** Summarizes current bioengineering strategies used to enhance the physiological relevance of hBOs in neurological disease (ND) research.

Optimization strategy	Construction method	Primary applications	Key advantages	Main limitations	References
Neuroimmune hBO models	Co-culture of hBOs with exogenous microglia	Investigating the role of microglia-specific gene mutations in ND	Microglia can reshape the immune microenvironment of hBOs	Limited microglial maturation and inconsistent integration	Abud et al., 2017; Song et al., 2019; Bejoy et al., 2019; Ao et al., 2021; Popova et al., 2021; Muffat et al., 2018; Abreu et al., 2018
	Integration of microglial progenitors into hBOs, matured within the hBO microenvironment	Exploring microglial development and its influence on neurodevelopment under normal and ND conditions	Reflects native-like microglial behavior in a human neural context	Time-consuming protocols and variable engraftment efficiency	Wörsdörfer et al., 2019; Sabate-Soler et al., 2022; Fagerlund et al., 2021
	Co-culture of microglial progenitors with neural progenitor cells (NPCs)	Studying microglia–NPC interactions in ND pathogenesis	Models early neuroimmune interactions relevant to disease onset	Simplified immune complexity and reduced spatial resolution	Xu et al., 2021
	Spontaneous differentiation of PSCs into hBOs containing microglia	Providing an accessible model for investigating neuroimmune mechanisms in ND	No external manipulation of microglia; highly integrated immune responses	Low microglial yield and lack of activation diversity	Ormel et al., 2018; Bodnar et al., 2021; Samudyata et al., 2022
hBO–rodent transplantation models	Transplantation of hBOs into healthy rodent brains	Studying gene function, neural development, and activity *in vivo* with a human genetic background	Enables *in vivo* analysis of hBO integration, activity, and disease modeling	Species mismatch, ethical concerns, and limited long-term viability	Shi et al., 2020; Daviaud et al., 2018; Kitahara et al., 2020; Dong et al., 2021; Revah et al., 2022
	Transplantation of hBOs into disease-model rodents	Evaluating the therapeutic efficacy of hBOs for ND treatment	Facilitates testing of transplantation-based therapies in ND	Invasive procedure and variability in host response	Wang Z. et al., 2020; Bao et al., 2021; Cao et al., 2023
Organoid assemblies	Fusion of brain region-specific organoids	Investigating neurodevelopment, neural circuitry, and ND-related interregional interactions	Enables study of multi-region and multi-organ dynamics	Fusion heterogeneity and poor reproducibility of connectivity	Birey et al., 2017; Bagley et al., 2017; Kasai et al., 2020; Miura et al., 2020
	Integration of hBOs with non-brain organoids	Modeling inter-organ communication in the context of ND	Simulates systemic influences on brain pathophysiology	Limited organ compatibility and functional synchronization	Zhu et al., 2023; Andersen et al., 2020; Son et al., 2022; Pereira et al., 2021; Workman et al., 2017

It highlights key construction methods, primary applications, advantages, limitations and representative studies for each approach, including neuroimmune models, rodent transplantation, and organoid assembly techniques.

### 5.1 Alzheimer’s disease (AD)

Alzheimer’s disease is a progressive neurodegenerative diseases (NDGD) primarily characterized by cognitive decline and behavioral disturbances. Its key pathological hallmarks include extracellular amyloid-beta (Aβ) plaque deposition and intracellular accumulation of hyperphosphorylated tau (P-Tau), ultimately leading to neurofibrillary tangles (Arvanitakis et al., 2019). Chen et al. (2021) demonstrated that hBOs exposed to serum from AD patients reproduced core AD pathologies, including elevated P-Tau expression, Aβ aggregation, disrupted neural networks, and synaptic degeneration. Similarly, Pavoni et al. (2018) showed that exogenous administration of Aβ42 to hBOs led to time-dependent Aβ accumulation and plaque formation. Mechanistic studies by Lee et al. (2022) revealed that Zika virus–induced activation of the PERK/eIF2α signaling pathway in hBOs could trigger AD-like pathological features. Notably, pharmacological inhibition of PERK significantly alleviated these abnormalities, offering valuable therapeutic insights (Lee et al., 2022). Zhao et al. (2020) demonstrated that hBOs derived from AD patients carrying the *APOE*ε4/ε4 genotype exhibited increased apoptosis, reduced synaptic integrity, and exacerbated tau phosphorylation compared to those from *APOE*ε3/ε3 individuals. Notably, isogenic conversion of *APOE4* to *APOE3* significantly attenuated these pathological phenotypes, supporting *APOE4*’s role in driving neurodegenerative processes in AD (Zhao et al., 2020). Perez-Corredor et al. (2024) applied CRISPR-Cas9 to convert the *APOE3*Ch allele to wild-type *APOE3* in hBOs, finding that *APOE3*Ch substantially reduced tau pathology in AD organoid models. Additionally, astrocytes and microglia derived from *APOE4*-genotyped hBOs demonstrated impaired Aβ42 uptake, while *APOE4*-to-*APOE3* conversion markedly improved these pathological features (Kim et al., 2015). Penney et al. (2020) highlighted that hBOs derived from iPSCs can successfully recapitulate key cellular dysfunctions observed in AD, including impaired neuron–glia interactions and AD-associated molecular phenotypes, underscoring the utility of hBO-based platforms in modeling complex neurodegenerative processes. Choi et al. (2014) established a 3D hBO model harboring familial AD mutations that faithfully recapitulated both extracellular amyloid-β plaque deposition and intracellular tau aggregation. Treatment with β- and γ-secretase inhibitors significantly reduced amyloid and tau pathologies, demonstrating the model’s potential for therapeutic screening (Choi et al., 2014).

### 5.2 Parkinson’s disease (PD)

Parkinson’s disease, the second most prevalent NDGD, is clinically characterized by bradykinesia, resting tremors, and muscular rigidity. Its pathological hallmarks include progressive dopaminergic neuron loss in the substantia nigra and the formation of Lewy bodies. Mohamed et al. detected α-synuclein aggregates in hBOs derived from *SNCA*-mutant cells, leading to dopaminergic neuronal degeneration (Mohamed et al., 2021). Kano et al. (2020) reported a significant reduction in astrocyte populations in PD-hBOs harboring *PRKN* mutations, mirroring neuropathological changes observed in PD patients with these variants. Furthermore, *LRRK2* mutations introduced into healthy PSCs successfully recapitulated hallmark PD features, including dopaminergic neuron loss and Lewy body formation (Zagare et al., 2022). Wulansari et al. (2021) demonstrated that *DNAJC6* mutations impaired *WNT–LMX1A* signaling, increased α-synuclein accumulation, and disrupted autophagy–lysosomal function in hBOs. Significantly, treatment of *PINK1*-mutant hBOs with 2-hydroxypropyl-β-cyclodextrin (2-HP-β-CD) improved mitochondrial function and neuronal autophagy, reducing dopaminergic neuron degeneration and necrosis. These findings suggest that 2-HP-β-CD may serve as a promising disease-modifying therapy for PD (Jarazo et al., 2022). Zheng et al. (2023) transplanted hBOs derived from healthy human cells into the striatum of immunodeficient PD model mice. The organoids successfully engrafted, matured, and significantly improved motor function, underscoring their potential for cell-replacement therapy in PD (Zheng et al., 2023). Additionally, CRISPR-generated *LRRK2*-knockout hBOs reproduced PD-related pathology, further confirming *LRRK2*’s pivotal role in PD pathogenesis (Kim et al., 2019).

### 5.3 Amyotrophic lateral sclerosis (ALS)

Amyotrophic lateral sclerosis is a progressive NDGD marked by the degeneration of both upper and lower motor neurons. Genetic studies have identified 42 ALS-associated genes, including *C9ORF72*, *ATXN2*, and TAR DNA-binding protein 43 (TDP-43). Pathogenic mechanisms include excitotoxicity, oxidative stress imbalance, and mitochondrial dysfunction (Brown and Al-Chalabi, 2017). Szebényi et al. (2021) developed an hBO slice culture model from iPSCs of *C9ORF72* ALS patients and identified early pathological features, including P62 accumulation in astroglia, poly(GA) dipeptide aggregates, DNA damage, and nuclear pyknosis in deep-layer neurons, which were partially rescued by treatment with the PERK inhibitor GSK2606414. Tamaki et al. (2023) reported that TDP-43 aggregates spread intercellularly within hBOs. This spread triggers astrocyte proliferation, DNA double-strand breaks, and cell death, which are hallmarks of ALS pathology. de Majo et al. (2023) demonstrated that *GRN*-deficient astrocytes induced TDP-43 hyperphosphorylation and misfolding in hBOs, a molecular signature of TDP-43 proteinopathy. Furthermore, co-culture models of hBOs and microglia have offered deeper insights into how glial cells, especially microglia and astrocytes, interact in ALS pathogenesis (Hong et al., 2023).

### 5.4 Autism spectrum disorders (ASD)

Autism spectrum disorders is a complex neurodevelopmental condition characterized by deficits in social communication, language impairments, and repetitive behaviors, with highly heterogeneous genetic and environmental etiologies. Zhang et al. (2020) reported that mutations in *RAB39B* led to increased hBO volume and excessive neural progenitor cell (NPC) proliferation, resulting in thickened SOX2^+^ ventricular zones and impaired neuronal differentiation. These abnormalities were attributed to hyperactivation of the PI3K-AKT-mTOR signaling pathway following *RAB39B* deletion (Zhang et al., 2020). Wang et al. utilized CRISPR-Cas9 to generate *CHD8*-deficient iPSCs, which were subsequently differentiated into hBOs. Their study revealed that *CHD8* regulates ASD-associated genes, such as *TCF4* and *AUTS2*, affecting Wnt/β-catenin signaling and GABAergic neuron differentiation–key processes implicated in ASD pathogenesis (Wang P. et al., 2017). Mariani et al. (2015) demonstrated that hBOs derived from ASD patients exhibited accelerated NPC cell cycle progression during early neurodevelopment, leading to overproduction of GABAergic neurons and resulting in an excitatory/inhibitory (E/I) imbalance. This phenotype was potentially driven by dysregulation of the *FOXG1* gene (Mariani et al., 2015). Schafer et al. (2019) analyzed patient-derived hBOs and identified asynchronous disruptions in gene regulatory networks during early NPC development, which prematurely promoted neuronal differentiation. Additionally, Marchetto et al. (2017) showed that aberrant regulation of the β-catenin/*BRN2* transcriptional axis resulted in synaptic transmission deficits and functional impairments in neuronal networks derived from ASD hBOs.

### 5.5 Achalasia-microcephaly syndrome (AMS)

Achalasia-microcephaly syndrome is a neurodevelopmental disorder (NDVD) characterized by imbalanced NPC proliferation and apoptosis, leading to reduced neuronal and glial populations and resulting in structural abnormalities of the brain. Key genes implicated in AMS pathogenesis include *NARS1*, *WDR62*, *CDK5RAP2*, and *CPAP*. Lancaster et al. (2013) were among the first to model AMS using hBOs derived from patient-specific iPSCs carrying *CDK5RAP2* mutations. These organoids exhibited key pathological features, including impaired NPC proliferation and premature neuronal differentiation. Moreover, by employing RNA interference and patient-derived iPSCs, hBOs were generated that recapitulated the core characteristics of microcephaly, such as disrupted progenitor zone organization and early neurogenesis, providing critical insights into the cellular mechanisms contributing to the reduced brain size observed in AMS patients (Lancaster et al., 2013). Wang L. et al. (2020) developed cortical hBOs from AMS patients with *NARS1* mutations and observed diminished proliferative capacity of radial glial cells (RGCs) and disrupted lineage specification of both RGCs and astrocytes. These findings underscore the critical role of *NARS1* in RGC regulation during brain development (Wang L. et al., 2020). Gabriel et al. (2016) used iPSC-derived hBOs to show that loss of *CPAP*, another AMS-associated gene, induced a premature shift from symmetric to asymmetric NPC division, ultimately impairing neurogenesis. Similarly, Zhang et al. (2019) modeled primary AMS using hBOs bearing *WDR62* mutations and reported defects in NPC cell cycle progression and reduced outer radial glia proliferation.

### 5.6 Epilepsy (EP) and brain tumors (BT)

Human brain organoids have emerged as powerful platforms for investigating the pathogenesis of EP and BT. Adeyeye et al. (2024) demonstrated the utility of integrating hBOs with microelectrode array (MEA) technology to study impaired plasticity and aberrant information processing in epileptic neural circuits. Brown et al. (2024) demonstrated that hBOs replicate key developmental and electrophysiological features of genetic epilepsies, including hyperexcitability dynamics and responsiveness to antiepileptic drugs, thus providing a physiologically relevant 3D model to investigate EP and screen therapeutic compounds. By engineering diverse hBO-based epilepsy models, researchers have explored mechanisms underlying EP and the interplay between neuronal firing patterns, cellular maturation, and subtype-specific vulnerability (Gross, 2022). Moreover, hBOs deficient in *CDKL5* successfully recapitulated epilepsy-related phenotypes. These models revealed that *CDKL5* mutations cause early-stage cortical neuron hyperexcitability, which is followed by late-stage hypoexcitability. Importantly, both hyperexcitability and hypoexcitability were reversed by pharmacological or gene therapy interventions (Negraes et al., 2021). In BT research, Linkous et al. (2019) developed a glioblastoma (GBM) model by co-culturing patient-derived glioblastoma stem cells (GSCs) with embryonic stem cells (ESCs) to generate GSC-hBOs. These GSCs exhibited deep tissue infiltration, proliferated within host tissue, and formed tumor-like masses that closely mimicked primary GBM pathology (Linkous et al., 2019). In studies of medulloblastoma (MB), Ballabio et al. (2020) demonstrated that *SMARCA4* suppresses the oncogenic activity of the *OTX2/MYC* axis in both patient-derived tissues and hBO models. Treatment with an *EZH2*-specific inhibitor significantly reduced *OTX2/MYC*-driven tumorigenesis in hBOs, underscoring the potential of hBOs for modeling genetic drivers and therapeutic responses in MB.

## 6 Conclusion and future perspectives

Human brain organoids represent powerful tools for modeling ND including AD, PD, ASD, ALS, AMS and others. By providing representative examples, this review highlights how hBOs are being applied to investigate disease-specific mechanisms and therapy. Compared to 2D cultures and animal models, hBOs better replicate the structural complexity and cellular diversity of the human brain, enhancing their translational relevance. However, several limitations persist, including limited vascularization, incomplete neuronal maturation, batch variability, lack of microglia and mature oligodendrocytes, and ethical concerns. While emerging technologies such as multi-omics integration, gene editing, and biomaterial engineering hold great promise, this mini-review does not provide comprehensive coverage of those aspects. Continued progress in standardization and bioengineering will be essential to overcome current challenges and unlock the full diagnostic and therapeutic potential of hBOs in neuroscience research.
